# The Surgical Diseases of the Genitourinary Tract, Venereal and Sexual Diseases

**Published:** 1905-06

**Authors:** 


### BOOK REVIEWS.

The Surgical Diseases of the Genitourinary Tract, Venereal and Sexual Diseases. A Textbook for Students and Practitioners. By G. Frank Lydston, M.D., Professor of the Surgical Diseases of the Genitourinary Organs and Syphilology in the Medical Department of the State University of Illinois. Second revised edition. Illustrated with 233 engravings and 7 colored plates. Pages xv.-1008. Philadelphia: F. A. Davis Company. 1904. (Cloth, $5.00 net; sheep or half russia, $6.00 net.)

Whatever the motives for a new edition of Lydston's work on genitourinary diseases,—whether the book's wide popularity has exhausted the first edition or whether the author with his well known strenuousness has insisted on keeping in the forefront, matters not; the fact remains that a second edition has been issued and with the author's thoroughness the contents have been brought up to the very gates of today. Lydston is nothing if not thorough and this commendable trait—which pray heaven may be emulated by more chesty if not better writers—is apparent everywhere.

Pity piled on pity it is that Dr. Lydston's publishers should be guilty of typographic crime in the mechanic production of the book; such for instance as allowing a half-page of print to slip its moorings and leave a chapter ending like the finish of a Chinese primer. However, the matter is there; so, little thought to the arrangement on the part of the printer. It is purely a matter of taste whether a man wears diamonds on his fingers or on his toes,—so long as he has the diamonds.

In reviewing other works of a purely textbook type in the past, regret has been expressed as politely as all regrets should be expressed in a kind-hearted review of a medical book where the intentions are good, that the same old shop worn cuts should have been used over and over again in these days of modern printing and illustration. Mention is made of this merely to accentuate the pleasure of the announcement that in Lydston's revised edition many new illustrations appear in place of the less eloquent plates which appeared in the first issue. In his introduction Dr. Lydston, a master of epigram, and a writer who dips his pen into the milk of human kindness only on special occasions, says: "Illustrations without a message have no place in a textbook," and those deaf and dumb cuts which were in the first book find no haven in the new edition.

All the nice things which were said of the previous issue of the work may be repeated and emphasised. Every phase of genitourinary work is covered in the author's original manner. There is no guess work as to what he means when he makes a statement, and one of the most refreshing things about his style, a style which is convincing and satisfying, is that he has the courage of his convictions and gives his readers the benefits of his own experiences without in the least slighting other investigators. The personal element is strongly accentuated throughout the book which, while it is scientific in all its aspects, has a chatty flavor which sharply contrasts with the unsatisfying brevity of some work; the top heavy weightiness of one other and the wearisome prolixity of still another writer, saturated with his subject and anxious to get it into a book. A man may be forgiven some of the unimportant shortcomings of literary style or scientific accuracy, who stands out strong and loyal to the memory of his master, and one of the striking features of the book is Lydston's worship at the shrine of Otis, especially marked in the chapter devoted to syphilis. While he acknowledges the possibility, yes, and the probability of the germ origin of syphilis, he frankly states that except for this he has not changed his views with regard to the disease since 1884. He refers to the somewhat famous disagreement between Otis, who regarded a minute cell as the carrier of infection, and Taylor, who flung himself into the limelight in defense of his own theory, misunderstanding and misinterpreting Otis's declaration which, at that time and in view of recent advances in the bacteriologic study of syphilis, was far more sensible than many of the latter day monstrosities of thought which have been given birth. The work of Lustgarten, Niesser, Klebs and others is given full credit, but there is no mention made of the investigations of Jullien, of Paris, or DeLisle, of New York, whose announcement to the Paris Academy of Medicine in 1901 of the discovery and demonstration of the bacillus of syphilis appears to have been overlooked if not ignored.

It is gratifying to find that a man of Lydston's force is taking a common sense position regarding the danger of urethral cocainisation. He explains sudden deaths after sounding and relatively unimportant urethral operations as due to an explosion of poisonous material which has gradually accumulated. Quite two years ago the writer of this review made the statement in the JOURNAL, that he could not find in genitourinary literature any absolute statement that death had been caused in any case by cocaine in the urethra; that those cases wherein death had occurred after the application of cocaine were undoubtedly due to other causes, which would have been found if searched for; and, while no attempt was made at that time to give an explanation of the deaths, it was suggested that the primary cause was the same which produced the urethral chill and fever. Lydston's explanation makes the whole matter much clearer and his position is defensible, which is a matter of importance and of congratulation. In gonorrhea Lydston stands as a defender of irrigation, in spite of the hundreds of get-well-quick cures which are worse in fact than the get-rich-quick schemes of the fakir.

Lydston's statements in this connection are corroborated by men who have had wide experience in the treatment of gonorrhea, and it will not be long before the progressive men of the rank and file of the profession will return to the so-called Janet method. The "rank" will continue to report marvelous cures of simple urethritis with some proprietary remedy as an almost miraculous cure of a severe gonorrhea, thus disregarding the fact that any gonorrhea is severe.

If any fault can be conscientiously found with Lydston's treatment of any genitourinary condition it is his advice to make a dorsal incision in the case of subpreputial chancroid. When this is done infection of the cut surfaces and the subsequent swelling will make it difficult to get at the lesion. It were better to advise two lateral incisions back to the corona; they give upper and lower flaps which make all parts of the glans easily accessible.

Aside from its value as a textbook, and it is much more valuable than could be shown in a review, the book is of greatest interest to students of the psychology of sex. Lydston gives much space to this subject and none of it is wasted; it is all instructive and enlightening. As it stands the book is one of the best on the subject of genitourinary diseases; for while there may be spots where it is opinionated, it is nowhere blindly egotistic, and best of all, it is helpful. N. W. W.

